# Evaluation of pharmacological activity of *Hibiscus tiliaceus*

**DOI:** 10.1186/s40064-016-2891-0

**Published:** 2016-07-29

**Authors:** S. M. Abdul-Awal, Sonia Nazmir, Sonia Nasrin, Tauhidur Rahman Nurunnabi, Shaikh Jamal Uddin

**Affiliations:** 1 Biotechnology and Genetic Engineering Discipline, Khulna University, Khulna, 9208 Bangladesh; 2 Statistics Canada, 9700 Jasper Avenue (Canada Place), Edmonton, AB T5J4C3 Canada; 3 Soil Science Discipline, Khulna University, Khulna, 9208 Bangladesh; 4 Pharmacy Discipline, Khulna University, Khulna, 9208 Bangladesh

**Keywords:** Analgesic, Antimicrobial, Bioactive, Cytotoxicity, Neuropharmacological

## Abstract

*Hibiscus tiliaceus*, locally known as Bhola was examined for phytochemical properties and its cytotoxic, antibacterial, analgesic and neuropharmacological activities using the ethanol extract of leaf and bark. The phytochemical analysis of the leaf extract indicated the presence of tannins, whereas bark extract indicated the presence of alkaloid, reducing sugar and tannins. A preliminary cytotoxicity of these extracts was determined by a simple and low cost assay using brine shrimp lethality. The leaf extract of the plant exhibited moderate cytotoxic effect (LC_50_: 20 µg/ml, LC_90_: 40 µg/ml) whereas the bark extract exhibited low cytotoxic effect (LC_50_: 50 µg/ml). In the analgesic test, the leaf extract showed comparatively high analgesic action than bark extract. There was no activity found in the leaf extract against the test bacterial strains, however bark extract exhibited a very little inhibitory effect on *Staphylococcus aureus* and *Staphylococcus epidermidis*. In the neuropharmacological test, the leaf and bark extract produced a decrease in both the time of onset of sleeping and the total sleeping time. The present study showed evidence that both leaf and bark extract of *H. tiliaceus* contain medicinally important bioactive compounds, thereby used as traditional medicine.

## Background

*Hibiscus tiliaceus* (Malvaceae) is a typical plant of tropical climates found in the regions of mangroves in significant quantities in Bangladesh as well as subtropical America, Africa, Asia, Australia, and throughout the Pacific islands (Rosa et al. 2006). The plant was used as a traditional medicine by the people for the treatment of fever, coughs and dry throat, ear infections, chest congestion, diarrhea, dysentery and typhoid (Shaikh et al. 2009; Ramproshad et al. 2012). The extract of *H. tiliaceus* was reported to have various interesting pharmacological activities such as antioxidant, anti-inflammatory, anthelmintic and antimicrobial activity (Tambe and Bhambar 2014; Ramproshad et al. 2012; Borhade et al. 2012; Narender et al. 2009). In Malaysia and Indonesia, leaves were used to cool fevers soothe coughs and remove phlegm, whereas fresh buds were chewed and swallowed for dry throat. Fresh bark soaked in water was used for chest congestion and during birth (Polynesians), whereas the inner bark (with sap) soaked in water was used for labor pains and rubbed on stomach. The slimy sap of the bark, branches and flower buds were used as a mild laxative or as a lubricant in childbirth or labor pains and rubbed on stomach (Melecchi et al. 2006; Petard 1986). Before children had teeth, the mothers chewed the buds and gave the chewed material to the children to swallow. The flowers were used to treat bronchitis due to their emollient properties (Konczak et al. 1995). The flowers are still used in head garlands (Hargreaves et al. 1970). An aqueous extract of wood and fresh flowers is a registered treatment for skin diseases. The young leaves are edible and eaten by the Polynesians. As the various parts of the plant have historically been used for pain, inflammation, neuronal diseases etc., a phytochemical group test was performed to identify the type of chemical constituents present in the ethanolic extracts of leaf and bark and their effect was analysed for cytotoxic, antibacterial, analgesic and neuropharmacological activities.

## Methods

### Preparation of plant extracts

Leaf and bark of *H. tiliaceus* were collected from the Sundarban (Bangladesh) and identified by expert from Forestry and Wood Technology Discipline, Khulna University, Bangladesh. These plant materials were washed with water and minced into small pieces. The small pieces were sun dried for 15 days and ground into a fine powder by grinder (capacitor start motor, Wuhu Motor Factory, China) and extracted with aqueous ethanol (90 %; v/v), being shaking and stirring occasionally for a period of 8 days. The whole mixture of leaf and bark was filtered through a piece of clean, white cotton material and Whatman filter paper. The crude extract (filtrate) was concentrated by evaporation of solvent using rotary evaporator. The crude extracts were weighed and stored into 4 °C for further experiments.

### Preliminary phytochemical screening

The freshly prepared crude extract of leaf and bark was subjected to preliminary phytochemical testing to identify the phytochemical constituents such as alkaloids, flavonoids, saponins, reducing sugar, tannins, and gum by using the standard phytochemical methods (Trease and Evans 1983; Ghani 1998).

### Brine shrimp lethality bioassay

Brine shrimp lethality bioassay (Meyer et al. 1982) was performed to see the cytotoxic effect of the leaf and bark extracts of *H. tiliaceus* on brine shrimp nauplii. The eggs of *Artemia salina* were hatched for 24 h at room temperature (25–30 °C) in sea water to obtain nauplii. The crude extract (500 mg) of leaf and bark was dissolved in a solution containing 6 ml DMSO and 4 ml seawater (stock concentration: 50 mg/ml), separately. Different amounts of the aqueous extract of leaf and bark (50, 100, 200, 400 and 800 µg/ml) were added into test tubes, separately containing 10 ml seawater and 10 brine shrimp nauplii. For the control test each tube contained 10 ml artificial seawater and 10 living nauplii. After 24 h the number of survived nauplii in each test tube was counted and the percentage of lethality of brine shrimp nauplii was calculated for each concentration of the extracts.

### Screening for in vitro antibacterial activity

The antibacterial activity was investigated using Gram-positive bacteria (*Staphylococcus aureus*, *Staphylococcus epidermidis*, *Staphylococcus saprophyticus*, *Streptococcus pyogenes*) and Gram negative bacteria (*Plesiomonas shigelloides*, *Shigella dysenteriae*, *Vibrio cholera*, *Salmonella typhii*, *Shigella flexneri*, *Shigella boydii*, *Shigella sonnei*, *Pseudomonas aeruginosa*) by agar diffusion method (Bauer et al. 1996; Ahmed et al. 2003). These microorganisms were collected from the Microbiology Lab. of Square Pharmaceutical Limited, Pabna, Bangladesh and then preserved in Plant Biotechnology Lab. of Biotechnology and Genetic Engineering Discipline, Khulna University, Khulna, Bangladesh. At first microorganisms were incubated overnight at 37 °C into nutrient agar slants. Small portion of the subculture was transferred into nutrient broth and incubated at 37 °C until the growth reached log phase. Wells on seeded agar plates were prepared by using a sterile cork borer (5 mm diameter). The ethanol extracts of leaf and bark (50 μl each, stock concentration: 10 μg/μl), methanol (50 μl, stock concentration: 10 μg/μl) and Gentamycin (30 μg) were poured on the wells for sample, blank and standard, respectively in each petri dish and then kept in refrigerator (4 °C) for 2 h and then transferred to incubator at 37 °C for 20 h. The antibacterial activity of the test agent was determined by measuring the diameter of zone of inhibition in term of millimeter with a transparent scale.

### Analgesic action assay

Young Swiss-albino mice (age: 4–5 weeks, average weight: 20–30 g) were used for the experiment. Analgesic activity of the aqueous extract of bark and leaf was tested by the model of acetic acid induced writhing in mice (Ahmed et al. 2004; White et al. 1964). Experimental animals were randomly selected and divided into four groups (I, II, III and IV). Each group is consisting of four mice and received a particular treatment i.e. control (water), positive control (Diclofenac-Na, dose: 25 mg/kg body weight) and the leaf and bark extracts (500 mg/kg body weight).

### Phenobarbitone induced sleeping time test

Fifteen mice were divided into three groups (I, II and III). Group I was the control group, Group II and Group III were the experimental groups. The experimental groups were administered orally with the leaf and bark extract (prepared by distilled water and Tween-80), separately at the doses 500 mg/kg body weight. The control group was supplied with distilled water containing 0.1 % Tween-80. A 30 min interval was given to assure proper absorption. Sodium phenobarbitone (25 mg/kg body weight) was administered intraperitonially to all the groups and the time of onset of sleep and total sleeping time was observed (Dandiya and Cullumbine 1959). The time for onset of sleep was the time taken for the loss of writhing reflex, whereas total sleeping time represents the time between the loss and regain of writhing reflex. The onset of sleep and total sleeping time were recorded for both control and experimental groups.

### Statistical analysis

The data obtained from this experiment was subjected to *t* test. In all cases, results were expressed as the mean ± standard error of mean. Significance level was evaluated from p value.

## Results

### Phytochemical properties

The results of phytochemical group test revealed that only tannins were present in the leaf extract, whereas alkaloids, reducing sugar and tannins were found in the bark extract (Table 1).

**Table 1:** Phytochemical constituents of leaf and bark extract of *H. tiliaceus*

Test for phytochemical group	Reagent	Results	
		Leaf	Bark
Alkaloids	Mayer’s test	–	+
	Dragendroff’s test	–	+
	Wagner’s test	–	+
	Hager’s test	–	+
Flavonoids	Shinoda’s test	–	–
Saponins	Frothing test	–	–
Reducing sugars	Fehling’s test	–	+
	Benedict’s test	–	+
	Combined reducing sugar test	–	+
Tannins	Ferric chloride test	+	+
	Potassium dichromate test	+	+
	Lead acetate test	+	+
Tests for Gums	Molish reagent and sulfuric acid test	–	+

+ Presence, − absence

### Cytotoxic activity

**Fig. 1:**
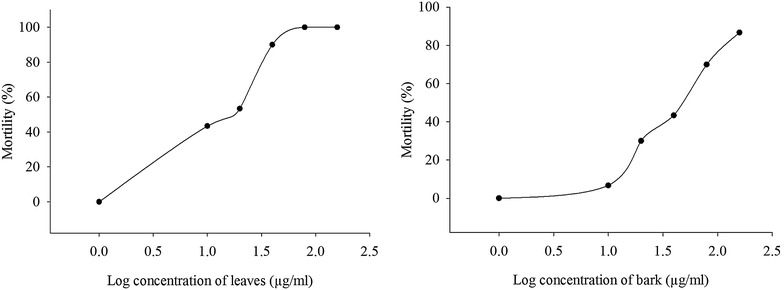
Brine Shrimp lethality activity of leaf and bark extract of *H. tiliaceus*. LC_50_ and LC_90_ concentrations were calculated by extrapolating of the graph

The crude extract of leaf and bark showed lethality indicating the presence of cytotoxic compounds in these extracts. Completely randomized design was followed during this bioassay (Kothari 1978). The mortality rate of brine shrimp was increased according to the increasing concentration of the leaf and bark extract (Fig. 1). An approximate linear correlation was found between percent mortality versus log concentration. The concentrations at which 50 % mortality (LC_50_) and 90 % mortality (LC_90_) of brine shrimp nauplii occurred were obtained by extrapolating 20 and 40 µg/ml, respectively for leaf (Fig. 1). The concentrations at which 50 % mortality (LC_50_) of brine shrimp nauplii occurred was obtained by extrapolating 50 µg/ml for bark (Fig. 1).

### Antibacterial activity

The leaf extract of *H. tiliaceus* showed no activity against supplied test bacterial strains, whereas bark extract showed shuttle inhibitory activity against *Staphylococcus aureus* and *Staphylococcus epidermidis* (Table 2).

**Table 2:** Antibacterial activity of leaf and bark extract of *H. tiliaceus*

Name of bacteria	Diameter zone of inhibition in mm		
	Gentamycin (30 μg/well)	Leaf extract (500 μg/well)	Bark extract (500 μg/well)
*Staphylococcus aureus*	23	–	4
*Staphylococcus epidermidis*	21	–	8
*Staphylococcus saprophyticus*	32	–	–
*Streptococcus pyogenes*	21	–	–
*Plesiomonasshigelloides*	24	–	–
*Shigelladysenteriae*	24	–	–
*Vibrio cholerae*	28	–	
*Salmonella typhii*	31	–	–
*Shigella flexneri*	21	–	–
*Shigella boydii*	23	–	–
*Shigella sonnei*	24	–	–
*Pseudomonas aeruginosa*	27	–	–

### Analgesic activity

The leaf & bark extract of *H. tiliaceus* produced 40.5 and 52.07 % protection or writhing inhibition in mice (oral dose: 500 mg/kg body weight of mice), respectively (Table 3). From the calculated p value it can be predicted with 95 % confidence that both extracts have significant analgesic effect (p < 0.05).

**Table 3:** Effect of leaf and bark extract of *H. tiliaceus* on acetic acid induced writhing in mice

Treatment (*n* = 4)	Dose (mg/kg)	No. of writhes	% Inhibition
Control	–	30.25 ± 4.13	–
Diclofenac sodium	25	18.00 ± 1.29*	59.50
*Hibiscus tiliaceus* leaves	500	15.75 ± 4.92*	40.5
*Hibiscus tiliaceus* barks	500	12.25 ± 4.69*	52.07

Values are mean ± SEM

* *p* < 0.05 versus control

### Phenobarbitone induce sleeping time test

The leaf and bark extract of *H. tiliaceus* produced a decrease in the time of onset of sleeping and total sleeping time. In control group the time of onset of sleep was 32.8 min, whereas the time of onset of sleep was 16.4 and 13.8 min for leaf and bark extract, respectively (Table 4). The total sleeping time was about 128.2, 133.4 and 101.4 min for the control, leaf and bark extract, respectively (Table 4).

**Table 4:** Effect of leaf and bark extract of *H. tiliaceus* on pentobarbitone-induced sleeping time in mice

Treatment (*n* = 5)	Dose (mg/kg)	Onset of sleep (min)	Duration of sleep (min)
Control	–	32.80 ± 7.03	128.20 ± 19.79
*Hibiscus tiliaceus* leaves	500	16.40 ± 1.03*	133.40 ± 13.87*
*Hibiscus tiliaceus* barks	500	13.80 ± 2.08*	101.40 ± 12.54*

Values are mean ± SEM

* *p* < 0.05 versus control

## Discussion

Most of the plants possess different kinds of secondary metabolites which are used as drugs, medicine or therapeutic agents for the treatment of different kinds of diseases. Medicinal plants hold an importance in the economy of any country as well as can bring a remarkable change in pharmaceutical sector. The phytochemical diversity of a medicinal plants include terpenoids, saponins, phenolics and phenyl propanoids, stilbenes, alkaloids, glucosinolates, hydrogen cyanide, indole and also elemental sulphur, the sole inorganic compound (Dhandapani and Sabna 2008). The phytochemical analysis of the leaf and bark extract of the *H. tiliaceus* showed the presence of different groups of secondary metabolites such as tannins, alkaloids, carbohydrates and reducing sugar (Table 1). All of them are known to possess medicinal activity as well as physiological activity (Sofowara 1993). The presence of these compounds may give credence to its local usage for the management of oxidative stress induced ailments. Tannins have been used traditionally for the treatment of diarrhea, hemorrhage and detoxification (Afolayan and Mabebie 2010; Okwu and Emenike 2006). The presence of tannins in leaf extracts may justify its traditional usage for the management of diarrhea. Alkaloids, comprising a large group of nitrogeneous compounds are widely used as cancer chemotherapeutic agents (Chabner and Hortwitz 1990; Noble 1990). Alkaloids also interfere with cell division, hence the presence of alkaloids in the bark extract could account for the brine shrimps lethality recorded in this study (Fig. 1). Alkaloids and saponins have a history of pharmacological effects for their analgesic and antispasmodic effects. These compounds have been used for the management of chest pain and arthritis among other diseases in South Africa (Edeoga et al. 2005; Njoku and Obi 2009). The crude extracts resulting in LC_50_ values <250 µg/ml are considered significantly active (Apu et al. 2010). The results indicate the ability of the plant extract to kill cancer cells in cell cultures, kill pests, and exert a wide range of pharmacologic effects (MacLaughin et al. 1991). Both leaf and bark extract could be the potential candidates for determination of antitumor and anticancer properties.

The bark extract showed inhibitory effect on *Staphylococcus aureus* and *Staphylococcus epidermidis* (Table 2) indicating the traditional use of this plant for ear infections, chest congestion and food poisoning (Shaikh et al. 2009; Wong et al. 2010; Ramproshad et al. 2012). It is a preliminary investigation for antimicrobial activity and further study with more bacterial strains should be performed for the precise scientific evidence. The leaf and bark extract of *H. tiliaceus* demonstrated significant analgesic activity (Table 3) and may be acting through inhibition of prostaglandin pathway and/or through peripheral pain mechanism. The result of the present study indicates that the crude extract of leaf and bark produced an alterations in general behavior pattern and both extracts produced a decrease in the time of onset of sleeping as well as total sleeping time (Table 4). It is possible that the extracts have no positive central depressant activity but may possess central nervous system (CNS) stimulating activity (Perez et al. 1998). However, more study should be performed for the isolation and characterization of the bioactive compound(s) and determination of the exact mechanism of action using different animal models.

## Conclusion

Pharmacological evaluation of *H. tiliaceus* leaf and bark extract showed cytotoxic, analgesic and neuropharmacological activities due to the presence of different active secondary metabolites in the extracts and perhaps some of these compounds may function in a synergistic manner. This is a preliminary investigation which may serve as a stepping stone for future research on the biological and pharmacological activities of *H. tiliaceus.*

## Abbreviations

LC: lethal concentration
CNS: central nervous system
